# Characterisation of the expression of P2X7 receptor, cancer stem cell markers and immunological mediators in human high-grade gliomas

**DOI:** 10.1186/s12868-025-00973-5

**Published:** 2025-10-01

**Authors:** Liyen K. Kan, Matthew Drill, Andrea Muscat, Paul Sanfilippo, Richard P. Sequeira, Padmakrishnan C. Jayakrishnan, Anh Vo, Nicholas C. Wong, Marian Todaro, Catriona McLean, Katherine J. Drummond, Martin Hunn, David A. Williams, Terence J. O’Brien, Mastura Monif

**Affiliations:** 1 Department of Neuroscience, School of Translational Medicine, Monash University, 99 Commercial Road, Melbourne, VIC 3004 Australia; 2 Monash Health Translation Precinct (MHTP) Medical Genomics Facility, Hudson Institute of Medical Research, 45 Kanooka Grove, Clayton, VIC 3168 Australia; 3 Department of Neurosurgery, The Alfred Hospital, 55 Commercial Road, Melbourne, VIC 3004 Australia; 4 Department of Neurology, The Royal Melbourne Hospital, 300 Grattan Street, Parkville, VIC 3052 Australia; 5 Department of Neurology, The Alfred Hospital, 55 Commercial Road, Melbourne, VIC 3004 Australia; 6 Victorian Brain Bank (VBB), The Florey Institute of Neuroscience and Mental Health, 30 Royal Parade, Parkville, VIC 3052 Australia; 7 Department of Anatomical Pathology, The Alfred, 55 Commercial Road, Melbourne, VIC 3004 Australia; 8 Department of Neurosurgery, The Royal Melbourne Hospital, 300 Grattan Street, Parkville, VIC 3052 Australia; 9 Department of Physiology, The University of Melbourne, Corner Grattan Street and Royal Parade, Parkville, VIC 3052 Australia

**Keywords:** Purinergic receptor, P2X7R, Glioblastoma, qPCR, Inflammation, Tumour microenvironment

## Abstract

**Introduction:**

Glioblastoma is the most aggressive primary brain cancer. It is considered an ‘immunologically cold’ tumour where immune infiltrates are polarised to drive immunosuppression—and therefore tumour proliferation. An important driver of neuroinflammation in glioma is the purinergic P2X7 receptor (P2X7R). While much of the complex glioma microenvironment has been characterised, studies expounding the associations between various cytokines/chemokines, immune cell markers, P2X7R expression and glioma stemness are lacking. Here we aimed to characterise the mRNA expression profiles of various tumour markers, and common ‘pro-‘ and ‘anti-tumour’ inflammatory mediators, and correlate this to P2X7R expression in human high-grade glioma samples compared to ‘healthy’ non-tumour post-mortem brain.

**Methods:**

High grade gliomas were collected from 34 patients undergoing routine tumour resection surgery and compared to 12 ‘healthy’ post-mortem controls. High throughput qPCR was performed on extracted RNA converted to cDNA to examine a custom-made panel of 38 tumour and immune related genes.

**Results:**

Markers of innate immunity including CD68, S100A9, HLADR, NLPR3, interleukin (IL) 1β, IL-6, TNFα and NF-κB were significantly increased in human derived glioblastoma samples compared to healthy control brain. P2X7R was also upregulated in the glioma microenvironment and its expression was linked to the expression of VEGFB, MMP9, PCNA, IL-4 and IL-8. The level of expression of P2X7R was not associated with overall survival in high grade gliomas.

**Discussion:**

Collectively, this study confirms the significant overexpression of P2X7R in human high-grade gliomas as well as highlights the presence of a multidirectional neuroinflammatory milieu in which both tumour-promoting and tumour-suppressive genes are overexpressed.

## Introduction

Gliomas are the most common and aggressive brain malignancy with poor prognoses and limited treatment options. There is a complex interplay between tumour cells and immune cells within the glioma microenvironment. In the absence of disease, the central nervous system (CNS) exists as a relatively immunoprivileged site protected by the blood-brain barrier (BBB) and parenchymal microglia. Gliomagenesis—fuelled by hypoxia, angiogenesis and tissue remodelling—leads to the disruption of BBB integrity, provoking a vicious neuroinflammatory response [1]. In gliomas, this neuroinflammation is rather paradoxical; it is a reaction to combat tumourigenesis yet can facilitate tumour progression. Hence, despite extensive immune infiltration, glioblastoma, the most lethal form of glioma, has been classically termed as an ‘immunologically cold’ tumour, where immune infiltrates are polarised to drive immunosuppression—and therefore tumour proliferation—by the collective tumour microenvironment [1].

Although the glioma microenvironment is predominantly ‘immunosuppressive’, neuroinflammation in gliomas is mediated by a combination of pro- and anti-tumourigenic factors and immune cells. Glioma progression is a consequence of immune cell and cytokine dysregulation upon exposure to the tumour microenvironment. The imbalance of inflammatory mediators, prolonged inflammatory responses, and the release of immunomodulatory factors by glioma cells induces severe tissue damage, resulting in a predominantly tumour-promoting immunosuppressive milieu [2]. Various inflammatory mediators that support tumour growth are upregulated within the glioma microenvironment. These include interleukin (IL)-1-beta (IL-1β), IL-4, IL-6, IL-8, IL-10, IL-13, transforming growth factor-beta (TGF-β), tumour necrosis factor-alpha (TNF-α), hypoxia-inducible factor-1 (HIF-1), epidermal growth factor receptor (EGFR), vascular endothelial growth factor (VEGF) and matrix metalloproteinase (MMP)-9 [3–7]. These mediators act by either stimulating glioma invasion and angiogenesis, inhibiting anti-tumour activity by immune cells, or promoting resistance to apoptosis and therapy [4]. Conversely, the expression of some inflammatory compounds expedites tumour suppression. Namely, interferons – interferon (IFN)-α, IFN-β and IFN-γ - are associated with glioblastoma cytotoxicity and improved survival [4]. These cytokines are essential for initiating cytotoxic T cell responses and antigen processing. Immune cell infiltrates in the glioma microenvironment have also been reported to have altered functions. For instance, factors released within the tumour niche from glioma cells themselves, are known to skew infiltrating microglia/macrophages into immunosuppressive phenotypes which weakens the immune response and aids in tumour cell survival. These recruited immune cells simultaneously secrete cytokines and growth factors that can stimulate glioma proliferation and angiogenesis, such as IL10 and VEGF [8, 9]. The inflammatory imbalance in favour of tumour growth is an obvious hurdle likely contributing to poor prognostic outcomes in glioblastoma. Further research is required to understand the overall inflammatory composition of the glioma microenvironment.

An important driver of neuroinflammation in glioma is the purinergic P2X7 receptor (P2X7R). In cancer, P2X7R can influence the tumour microenvironment via the neuroinflammatory cascade and serve direct roles in tumour cell proliferation. Indeed, P2X7R can be activated in the glioma microenvironment in response to neuroinflammation, neuronal cell loss from tumour invasion, hypoxia and heightened release of extracellular ATP [10]. The receptor therefore can exacerbate gliomagenesis by promoting the release of various cytokines/chemokines and infiltration of immune cells which can facilitate a pro-tumour environment [10, 11]. However, the precise roles of P2X7R in high-grade gliomas remain unclear. There are also prognostic associations with P2X7R expression in some cancers, with varying levels of receptor expression correlating to tumour grade and invasiveness [12, 13]. For example, in both colorectal cancer and neuroblastoma, increased P2X7R expression was associated with worse prognosis and lower survival [12, 13]. However, limited research has been done to investigate the relationship between P2X7R expression and glioblastoma prognosis.

The expression of the inflammatory mediators in glioblastoma are well characterised, but our understanding of the overall glioma microenvironment requires further clarification. Few studies have investigated the expression of components that constitute the entire tumour microenvironment with reference to ‘normal’ human brain, particularly in terms of the associations between various tumour-promoting and tumour-suppressing mediators, immune cells, P2X7R, and glioma malignancy markers. Illuminating this would further contribute to the current knowledge of the multi-dimensional glioma milieu.

Here, the mRNA expression profiles of various tumour markers, and common ‘tumour-promoting’ and ‘tumour-suppressing’ mediators were characterised and correlated with P2X7R expression, in both human high-grade glioma samples and “healthy” non-tumour post-mortem brain. The markers selected represent the most investigated and pathophysiologically relevant markers in the context of glioblastoma. This study also elucidated whether P2X7R expression levels are associated with survival outcomes of patients with high-grade gliomas. Characterising the inflammatory profile in glioblastoma and its relationship to P2X7R would further delineate the role of P2X7R in glioma as promoting pro- or anti-inflammatory cytokine release, reveal possible therapeutic targets and potentially contribute to the current glioma biomarker pool.

## Materials and methods

### Collection of high-grade glioma samples and healthy brain tissue

Human high-grade glioma samples were collected from 34 patients (mean age: 54.9 ± 13.8 years; female: 13; male: 21; diagnoses: 30 glioblastoma (IDH WT), 4 WHO grade 3 glioma (IDH mutant)) undergoing routine tumour resection surgery at the Royal Melbourne Hospital (RMH) and The Alfred, in collaboration with the Alfred Brain Tumour Biobank (Melbourne, Australia). All glioma samples were diagnosed by a certified pathologist. Tumour samples were immediately snap-frozen and stored in liquid nitrogen at − 80 °C until RNA extraction to ensure sample integrity. Control samples were snap-frozen post-mortem ‘normal’ brain tissue from twelve individuals (mean age: 54.4 ± 7.4 years; female: 4; male: 8) and were received from the Victorian Brain Bank (HREC Alfred Health 729/19). Controls were stored at − 80 °C until use in RNA extraction.

### mRNA extraction and cDNA synthesis

mRNA was extracted from glioma and control brain tissue using the QIAGEN RNeasy^®^ Mini Kit, according to the manufacturer’s instructions (cat no. 74136). For each sample, 30 mg of frozen tissue was homogenised in manufacturer-supplied buffer containing 1% β-Mercaptoethanol with a blunt 20-gauge needle equipped to an RNase-free syringe. Samples were also subjected to on-column DNase digestion using the QIAGEN RNase-Free DNase Set, as per the manufacturer’s protocol (cat no. 79254). mRNA purity (260/280 ratio) was assessed with the NanoDrop™ Lite spectrophotometer (Thermo Fisher, cat no. ND-LITE-PR) prior to cDNA synthesis. Tumour and control cDNA were synthesised from 1 µg mRNA with the Roche Transcriptor First Strand cDNA Synthesis Kit, according to the manufacturer’s instructions (cat no. 04897030001). A combination of both anchored-oligo(dT)_18_ primers and random hexamer primers were used for cDNA synthesis.

### Quantitative PCR (qPCR)

This study utilised the BioMark system from Fluidigm on their 48.48 Dynamic Array Integrated Fluidic Circuits (IFC) to analyse a panel of 38 tumour- and immune-related genes against 10 housekeeping genes (Table 1). The qPCR was performed in collaboration with the Monash Health Translation Precinct (MHTP) Medical Genomics Facility (Melbourne, Australia). The selected genes were grouped into the following functional categories: P2X7R, tumour classification and glioma cell stemness markers (*GFAP*, *IDH1*, *IDH2*, *NANOG*, *SOX2*, *SOX2-OT*, *CD133*), ‘tumour-promoting’ mediators (*IL-1β*, *IL-4*, *IL-6*, *IL-8*, *IL-10*, *IL-13*, *TGF-β*, T*NF*, *VEGFA*, *VEGFB*, *EGFR*, *MMP-9*, *HIF-1α*, *NF-κB*, *PCNA*), ‘tumour-suppressing’ mediators (*IFN-α*, *IFN-β*, *IFN-γ*, *IL-2*, *IL-12A*, *IL-12B*) and immune cell markers (*CD68*, *CD45*, *ARG1*, *CD14*, *S100A8*, *S100A9*, *HLADR*, *FOXP3*, *NLRP3*). The housekeeping genes were *Actb*, *FBXW7*, *HPRT1*, *YWHAZ*, *CYC1*, *SDHA*, *TBP*, *GAPDH*, *UBC* and *18s rRNA*. One Taqman assay − 20X forward and reverse primer and probe mixes at a concentration of 18µM and 4µM, respectively – was selected for each gene. qPCR using *SYBR GAPDH* was conducted for all samples as a quality control check prior to loading onto the 48.48 gene expression IFC. All samples underwent a 14-cycle preamplification step, as detailed by Fluidigm (Quick Reference PN 100–5876 B1). For the 48.48 Dynamic Array IFC, 5 µL of each assay at 10X was added to each assay inlet port, and 5 µL of sample (diluted 1:5 in TE buffer) was added to each sample inlet port as per the Chip Pipetting Map (Quick Reference PN 68000089 H1). Data was analysed with the Fluidigm Real-Time PCR analysis software (V4.1.1). Raw cycle threshold (Ct) values were normalised to the housekeeping genes and converted into relative gene expression (fold change; FC) to the ‘healthy controls’ non-tumour human samples using the 2^− ΔΔCt^ method. The FC values were transformed into log2 FC (LFC) values for statistical analyses.

**Table 1:** List of Taqman assay IDs for selected genes quantified via high throughput qPCR

Gene	Taqman assay				Gene			Taqman assay		
Glioma cell stemness markers					MMP-9			Hs00234579_m1		
GFAP	Hs00909233_m1				HIF-1α			Hs00153153_m1		
IDH1	Hs01855675_s1				NF-κB			Hs00765730_m1		
IDH2	Hs00158033_m1				PCNA			Hs00427214_g1		
NANOG	Hs02387400_g1				Immune cell markers					
SOX2	Hs01053049_s1				P2X7R			Hs00175721_m1		
SOX2-OT	Hs00415716_m1				CD68			Hs02836816_g1		
CD133	Hs01009257_m1				CD45			Hs04189704_m1		
‘Tumour-suppressing’ mediators					ARG1			Hs00968979_m1		
IFN-α	Hs00855471_g1				CD14			Hs02621496_s1		
IFN-β	Hs01077958_s1				S100A8			Hs00374264_g1		
IFN-γ	Hs00989291_m1				S100A9			Hs00610058_m1		
IL-2	Hs00174114_m1				HLADR			Hs00219578_m1		
IL-12A	Hs01073447_m1				FOXP3			Hs01085834_m1		
IL-12B	Hs01011518_m1				NLRP3			Hs00918082_m1		
‘Tumour-promoting’ mediators					Housekeeping genes					
IL-1β	Hs01555410_m1				Actb			Hs99999903_m1		
IL-4	Hs00174122_m1				FBXW7			Hs00217794_m1		
IL-6	Hs00985639_m1				HPRT1			Hs02800695_m1		
IL-8	Hs00174103_m1				YWHAZ			Hs03044281_g1		
IL-10	Hs00961622_m1				CYC1			Hs00357718_g1		
IL-13	Hs00174379_m1				SDHA			Hs00188166_m1		
TGF-β	Hs00998133_m1				TBP			Hs00427620_m1		
TNF	Hs01113624_g1				GAPDH			Hs99999905_m1		
VEGFA	Hs00900055_m1				UBC			Hs00824723_m1		
VEGFB	Hs00173634_m1				18s rRNA			Hs03928985_g1		
EGFR	Hs01076078_m1									

### Immunohistochemistry

Paraffin-embedded glioblastoma sections were obtained from The Department of Pathology at Alfred Hospital for immunohistochemical analysis. Immunohistochemical staining was performed by the Monash Histology Platform, Department of Anatomy and Developmental Biology, Monash University. Briefly, sections were immersed in Dako retrieval solution for 30 min at 98 °C (cat no. S1699) and washed in duplicate with EnVision FLEX Wash Buffer (Dako, cat no. K8000) for 5 min at room temperature. Sections were blocked with Dako REAL Peroxidase-Blocking Solution (cat no. S2023) and subsequently protein block (Dako, cat no. X0909) for 10 min each. Slides were rinsed with EnVision FLEX Wash Buffer between each block step. Sections were stained for 1 h with primary rabbit anti-P2X7R polyclonal antibody (Sigma Aldrich, cat no. P8232, RRID: AB_261204; 1:160) or primary rabbit anti-GFAP polyclonal antibody (Dako, cat no. Z0334, RRID: AB_10013382; 1:3000), and washed for 5 min with EnVision FLEX Wash Buffer. Secondary staining was performed via immersion with goat anti-rabbit-HRP antibody (Dako, cat no. K4003, RRID: AB_2630375) for 30 min. Sections were subsequently washed in triplicate and stained for 10 min with EnVision FLEX DAB+ Substrate Chromogen System (Dako, cat no. GV825). Sections were stained for 5 min with haematoxylin (Dako, cat no. S3301), and rinsed twice with distilled water.

### Statistical analysis

All statistical analyses were conducted on GraphPad Prism (version 9). Unpaired t tests with Welch’s correction were used to compare the LFC values between tumour samples and controls. The minimum necessary sample size was determined through power testing and study design, though overall sample size was limited by availability of samples. Data were expressed as mean LFC ± SEM and the 95% confidence interval (CI). To assess the relationship between P2X7R and other genes of interest, a Pearson’s correlation test was used followed by a simple linear regression, with P2X7R as the independent variable. For survival analyses, a Kaplan-Meier analysis was performed with P2X7R as a categorical variable, with the outcome being overall survival (measured from the date of surgery to the date of death or last follow-up). P2X7R expression levels were categorised as either ‘low’ or ‘high’, based on values below or above the median, respectively. Differences in overall survival between groups were calculated using the log-rank test. A multivariable Cox proportional-hazards regression was also performed to assess the effect P2X7R on overall survival, with patient age and sex as additional variables. The hazard ratio (HR) and its respective 95% CI and p-value were reported. The significance level for all analyses was set at *p* < 0.05.

## Results

### The expression of various glioma classification and glioma cell stemness markers in the patient cohort relative to healthy controls

**Fig. 1:**
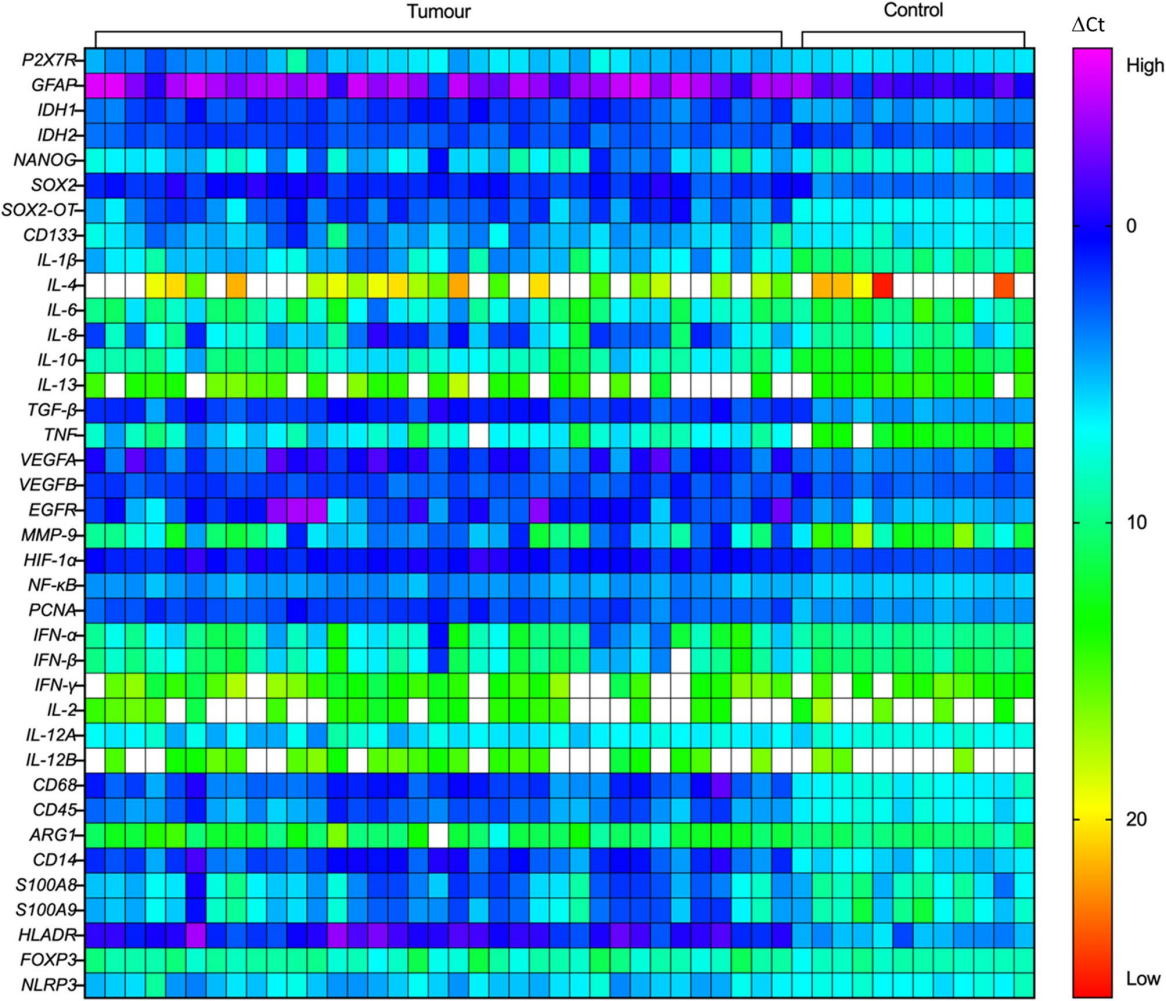
Heatmap of the ΔCt values of selected genes including P2X7 receptor (P2X7R), and genes involved in tumour classification and glioma cell stemness, immunity and inflammation in human high-grade glioma samples, compared to non-tumour post-mortem brain controls. Gene expression data was obtained from high throughput qPCR analyses using the Biomark 48.48 Dynamic Array Integrated Fluidic Circuits (IFC) system from Fluidigm. 38 genes of interest (GOI) and 10 housekeeping genes (not displayed) from 34 high-grade glioma samples and 12 non-tumour post-mortem brain specimens were included. Values are ΔCt values of each GOI normalised to the housekeeping genes. Lower ΔCt values correspond to higher gene expression

**Fig. 2:**
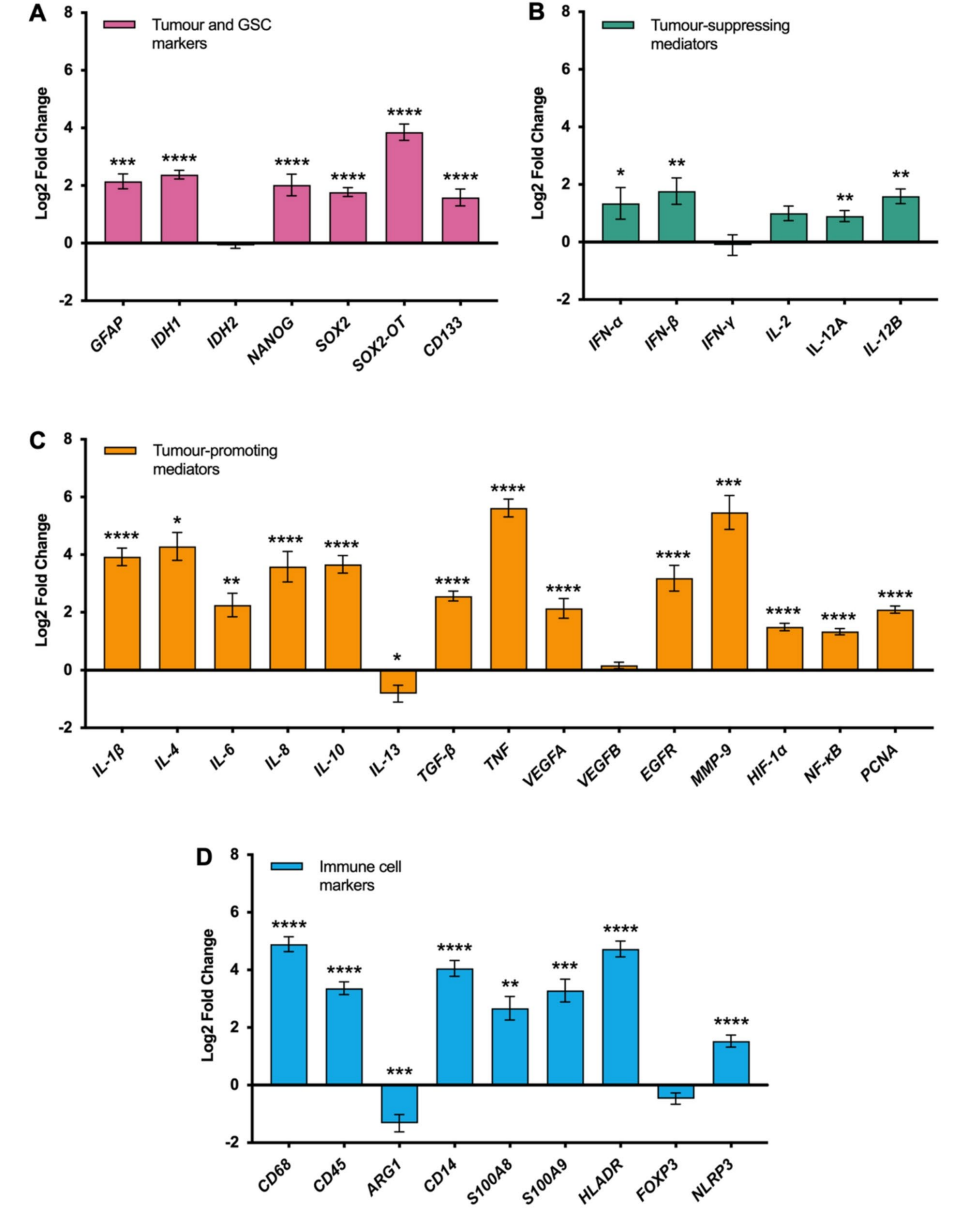
Relative expression of selected genes in human high-grade glioma samples compared to non-tumour post-mortem brain tissue controls. Gene expression data was obtained from high throughput qPCR analyses using the Biomark 48.48 Dynamic Array Integrated Fluidic Circuits (IFC) system from Fluidigm. 38 genes of interest and 10 housekeeping genes from 34 high-grade glioma samples and 12 non-tumour post-mortem brain specimens were included. The fold change (FC) in gene expression relative to controls was calculated using the 2^− ΔΔCt^ method for qPCR analysis. Data were transformed into log2 FC (LFC). Differences in LFC of tumours compared to the control were assessed with an unpaired t test: *p < 0.05, **p < 0.01, ***p < 0.001, ****p < 0.0001. Data are represented as mean LFC ± SEM

As a first step, the mRNA expression of various glioma and cancer cell stemness markers were characterised in the patient cohort of 30 high-grade glioma and 12 control brain samples. qPCR analysis of 38 genes was investigated, including the glioma cell marker, *GFAP*; prognostic glioma classification markers, *IDH1/2*; and glioma cell stemness markers, *NANOG*, *SOX2*, *SOX2-OT* and *CD133* (Fig. 1). The FC in gene expression of each marker in tumour samples relative to healthy controls was determined using the 2^− ΔΔCt^ method normalised to the average of 7 housekeeping genes (*YWHAZ*, *CYC1*, *SDHA*, *TBP*, *GAPDH*, *UBC* and *18s RNA*). *Actb*, *FBXW7* and *HPRT1* were differentially expressed between glioma samples and non-tumour controls and were therefore excluded as housekeeping genes. Tumour samples had significant upregulation of GFAP (4.44-fold increase, mean LFC = 2.16 ± 0.26; *p* = 0.0002, 95% CI = 1.14 to 3.15), IDH1 (5.21-fold increase, LFC = 2.38 ± 0.15; *p* < 0.0001, 95% CI = 1.87 to 2.89), NANOG (4.06-fold increase, mean LFC = 2.02 ± 0.37; *p* < 0.0001, 95% CI = 1.13 to 2.91), SOX2 (3.42-fold increase, mean LFC = 1.77 ± 0.15; *p* < 0.0001, 95% CI = 1.09 to 2.45), SOX2-OT (14.47-fold increase, mean LFC = 3.86 ± 0.28; *p* < 0.0001, 95% CI = 3.26 to 4.45), and CD133 (3.01-fold increase, mean LFC = 1.59 ± 0.29; *p* < 0.0001, 95% CI = 0.89 to 2.30). IDH2 levels were not significantly upregulated in glioma samples (Fig. 2A). Collectively, there was an increase in all of the above glioma and glioma stemness markers in tumour samples of the patient cohort, except for IDH2.

### Inflammatory mediators are upregulated in the glioma microenvironment in favour of gliomagenesis

The overall phenotype of the inflammatory glioma milieu was characterised by quantifying the gene expression profiles of various mediators that are commonly denoted as either promoters or suppressors of gliomagenesis. The selected tumour-promoting genes were: *IL-1β*,* IL-4*,* IL-6*,* IL-8*,* IL-10*,* IL-13*,* TGF-β*,* TNF*,* VEGFA*,* VEGFB*,* EGFR*,* MMP-9*,* HIF-1α*,* NF-κB*, and *PCNA*. The tumour-suppressing mediators measured were *IFN-α*,* IFN-β*,* IFN-γ*,* IL-2*,* IL-12A* and *IL-12B*. Tumour-promoting mediators were significantly upregulated in the glioma microenvironment, relative to ‘healthy’ non-tumour controls (Fig. 2C). Of note, *TNF* and *MMP-9* were the most upregulated pro-tumour genes, with expression in tumours being over 40-fold higher than the controls – *TNF*: 49.28-fold increase, mean LFC = 5.62 ± 0.31, *p* < 0.0001, 95% CI = 4.81 to 6.44; *MMP-9*: 44.26-fold increase, mean LFC = 5.468 ± 0.59, *p* = 0.0002, 95% CI = 3.03 to 7.90. Other overexpressed tumour-trophic genes included: *IL-1β* (15.22-fold increase, mean LFC = 3.93 ± 0.30; *p* < 0.0001, 95% CI = 2.99 to 4.87), *IL-4* (19.51-fold increase, LFC = 4.29 ± 0.48; *p* = 0.01, 95% CI = 1.41 to 7.17), IL-6 (4.77-fold increase, mean LFC = 2.26 ± 0.408; *p* < 0.0001, 95% CI = 0.81 to 3.70), *IL-8* (12-fold increase, mean LFC = 3.59 ± 0.53; *p* < 0.0001, 95% CI = 2.16 to 5.01), *IL-10* (12.69-fold increase, mean LFC = 3.67 ± 0.30; *p* < 0.0001, 95% CI = 2.74 to 4.59), *TGF-β* (5.92-fold increase, mean LFC = 2.57 ± 0.17; *p* < 0.0001, 95% CI = 1.91 to 3.22), *VEGFA* (4.40-fold increase, mean LFC = 2.14 ± 0.34; *p* < 0.0001, 95% CI = 1.32 to 2.95), *EGFR* (9.09-fold increase, LFC = 3.18 ± 0.45; *p* < 0.0001, 95% CI = 2.08 to 4.29), *HIF-1α* (2.81-fold increase, mean LFC = 1.49 ± 0.13; *p* < 0.0001, 95% CI = 1.12 to 1.86), *NF-κB* (2.52-fold increase, mean LFC = 1.33 ± 0.11; *p* < 0.0001, 95% CI = 1.05 to 1.62), and *PCNA* (4.28-fold increase, mean LFC = 2.10 ± 0.13; *p* < 0.0001, 95% CI = 1.70 to 2.50). There was interestingly lower *IL-13* expression in tumour samples relative to healthy brain (1.76-fold decrease, mean LFC=-0.81 ± 0.29; *p* = 0.04, 95% CI=-1.58 to -0.04). No significant change in *VEGFB* expression was observed. *p* = 0.54, 95% CI=-0.4 to 0.74).

Several mediators that promote glioma cell death were also upregulated in the glioma microenvironment, compared to normal brain (Fig. 2B). These included: *IFN-α* (2.54-fold increase, mean LFC = 1.35 ± 0.55; *p* = 0.02, 95% CI = 0.21 to 2.48), *IFN-β* (3.41-fold increase, LFC = 1.77 ± 0.46; *p* = 0.003, 95% CI = 0.651 to 2.89), *IL-12A* (1.87-fold increase, mean LFC = 0.90 ± 0.19; *p* = 0.002, 95% CI = 0.35 to 1.45), and *IL-12B* (3.02-fold increase, mean LFC = 1.59 ± 0.25; *p* = 0.007, 95% CI = 0.64 to 2.55). There were no significant increases in *IFN-γ* (*p* = 0.84, 95% CI=-1.14 to 0.93) or *IL-2* (*p* = 0.31, 95% CI=-1.32 to 3.33) gene expression.

While there was overexpression of both pro-tumour and anti-tumour mediators in the glioma microenvironment, pro-tumour mediators were more substantially upregulated. Hence, these data indicate that there is significant dysregulation of inflammatory mediators in high-grade gliomas, with conventionally described ‘immunostimulatory’ mediators highly upregulated in the tumour microenvironment.

### Immune cell infiltration in the glioma microenvironment

Immune cell infiltrates directly mediate the inflammatory response against gliomas as they both secrete cytokines and chemokines that exert differential effects in the glioma microenvironment, and also present tumour antigens to initiate anti-tumour responses. However, interactions with factors in the tumour milieu can influence immune cell activity, causing some cell types to deviate from their usual functions and ultimately work in favour of tumour proliferation. Hence, the presence of major immune cell infiltrates and their functional states was characterised by quantifying gene expression of various phenotypic immune cell markers: *CD68*,* CD45*,* ARG1*,* CD14*,* S100A8/9*,* HLADR*,* FOXP3 and NLRP3* (Fig. 2D). The most upregulated markers in tumour samples were *HLADR* (26.54-fold increase, mean LFC = 4.73 ± 0.27; *p* < 0.0001, 95% CI = 3.88 to 5.58), *CD68* (29.82-fold increase, mean LFC = 4.90 ± 0.26; *p* < 0.0001, 95% CI = 4.23 to 5.57) and *CD14* (16.61-fold increase, mean LFC = 4.05 ± 0.28; *p* < 0.0001, 95% CI = 3.37 to 4.74), correlating to increased monocyte and macrophage/microglia infiltration (*CD68* and *CD14*), and antigen presentation (*HLADR*). General inflammatory immune cell markers, CD45 (LFC = 3.37  ±  0.22, *p* < 0.0001, 95% CI = 2.78 to 3.96) and NLRP3 (LFC = 1.52  ±  0.21,, *p* < 0.0001, 95% CI = 0.95 to 2.11) were also significantly upregulated in glioma samples. Significantly higher gene expression levels of the S100A8/9 heterodimer: *S100A8*: 6.36-fold increase (LFC = 2.67 ± 0.41p = 0.002, 95% CI = 1.08 to 4.26); *S100A9*: 9.75-fold increase (mean LFC = 3.29 ± 0.40, *p* = 0.0005, 95% CI = 1.65 to 4.93), which are commonly associated with neutrophils and monocytes were also observed in high-grade glioma specimens. Surprisingly, there was a reduction in *ARG1* gene expression in tumour samples (2.50-fold decrease, mean LFC = −1.32 ± 0.30; *p* = 0.0008, 95% CI = −2.06 to −0.58), correlating to decreased levels of immunosuppressive ‘M2’ tumour-associated microglia/macrophages (TAMs). These results are supportive of amplified inflammatory immune cell infiltration in the glioma microenvironment, particularly monocytes and macrophages.

### P2X7R expression is not associated with overall survival in high-grade glioma

**Fig. 3:**
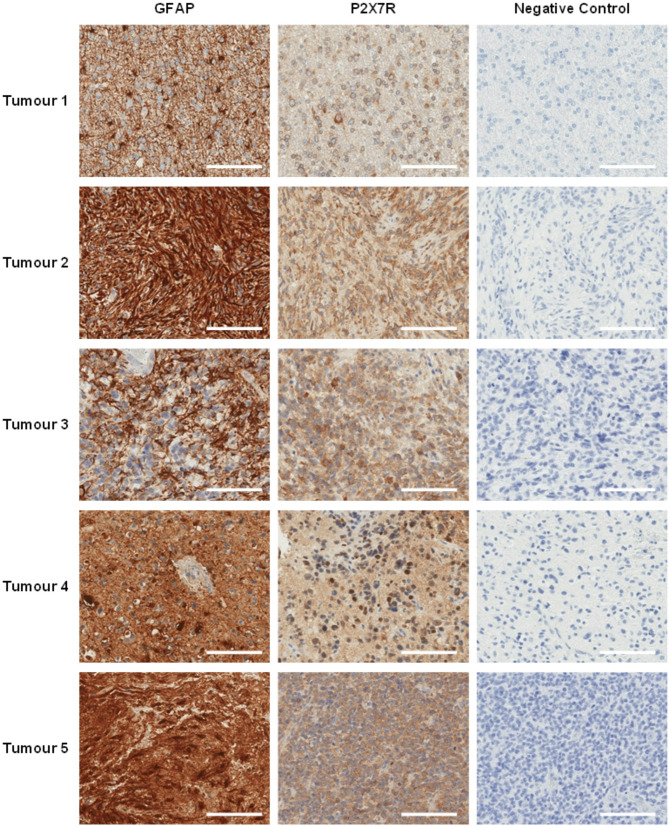
P2X7 receptor (P2X7R) and glial fibrillary acidic protein (GFAP) expression in human glioblastoma tissue. Paraffin-embedded human glioblastoma tissue sections (n = 5 patients) were stained with primary rabbit anti-P2X7R or anti-GFAP, followed by secondary goat anti-rabbit-horse radish peroxidase (HRP) and 3,3’-diaminobenzidine (DAB). Brightfield images are at 20X magnification. Scale bar: 100 μm

**Fig. 4:**
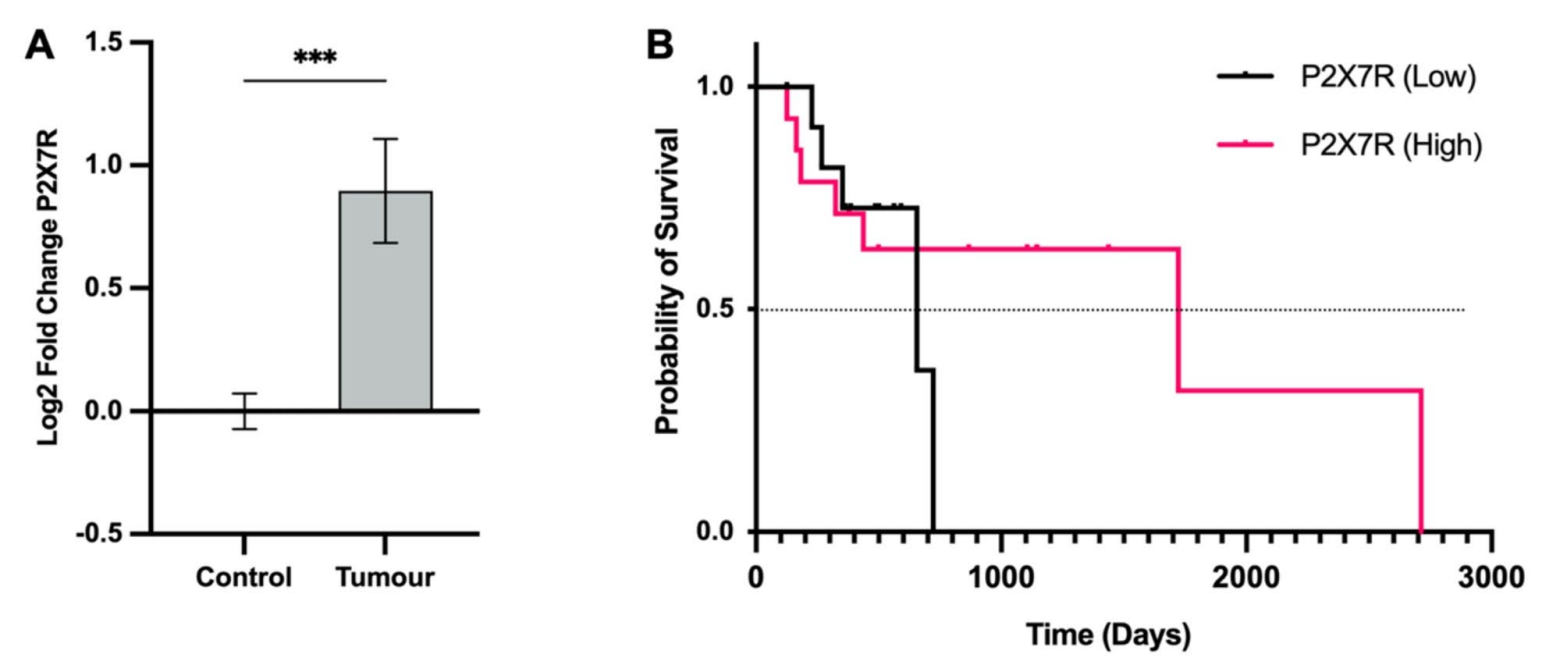
**A** Relative expression of P2X7 receptor (P2X7R) in human high-grade glioma samples compared to non-tumour post-mortem brain tissue controls. Gene expression data was obtained from high throughput qPCR analyses using the Biomark 48.48 Dynamic Array Integrated Fluidic Circuits (IFC) system from Fluidigm. The fold change (FC) in gene expression relative to control was calculated using the 2^− ΔΔCt^ method for qPCR analysis. Data were transformed into log2 FC (LFC). Differences in LFC of tumours compared to the control were assessed with an unpaired t test: ***p = 0.0003. Data are represented as mean LFC ± SEM. **B** Kaplan-Meier survival curves of high-grade glioma patients with low or high P2X7R expression. Data on overall survival (OS) were obtained from 29 high-grade glioma patients. OS was calculated as the number of days from the date of tumour resection until death or last follow-up. P2X7R expression was categorised as either low or high, based on values below or above the median, respectively. Survival curves between P2X7R-low and P2X7R-high groups were not significantly different: χ^2^(df:1) = 0.157, *p* = 0.692

Immunohistochemical staining was conducted on paraffin-embedded human glioblastoma tissue slices to demonstrate representative P2X7R expression in tumour tissue (GFAP+) obtained from 5 glioblastoma patients (Fig. 3). High throughput focused microarray analysis was utilised to quantify the expression of P2X7R in human high-grade glioma samples relative to non-tumour brain (Fig. 4A). Results demonstrated a significant 1.86-fold increase in P2X7R gene expression in tumour tissue of patients with high-grade gliomas, compared to post-mortem brain controls (mean LFC = 0.90 ± 0.21; *p* = 0.0003, 95% CI = 0.44 to 1.35).

P2X7R expression was subsequently investigated as a potential prognostic marker for high-grade gliomas. A survival analyses was conducted using both the Kaplan-Meier method and the Cox proportional hazards regression model to assess the effect of P2X7R expression on overall survival using the date of tumour resection to the date of death or last follow-up. For the Kaplan-Meier analysis, P2X7R expression was categorised into either P2X7R-low or P2X7R-high, based on expression levels below or above the median, respectively. The LFC in P2X7R expression was utilised as a continuous variable for the Cox proportional hazards regression, with patient age and sex considered. Overall survival data was collected from 29 patients diagnosed with high-grade glioma at RMH and The Alfred Hospital. A log-rank test of the differences in Kaplan-Meier survival distributions of patients with low and high P2X7R expression yielded no statistically significant differences between each group (χ^2^ (df:1) = 0.157, *p* = 0.692) (Fig. 4B). Similarly, results from the Cox proportional hazards regression demonstrated no significant association between P2X7R expression and overall survival, although increasing age was associated with a 0.6% increased risk of death per year - P2X7R: HR = 1.23, 95% CI = 0.81 to 1.87, *p* = 0.34; age: HR = 1.06, 95% CI = 1.01 to 1.11, *p* = 0.02; sex: HR = 1.49, 95% CI = 0.46 to 4.78, *p* = 0.50 (Table 2). Hence, there is likely no relationship between P2X7R expression and overall patient survival in high-grade gliomas.

**Table 2:** Multivariable Cox proportional-hazards regression assessing the relationship between P2X7 receptor (P2X7R) expression and overall survival of high-grade glioma

Variable	Hazard ratio (HR)	95% CI of HR	*p*-value
P2X7R	1.23	0.81, 1.87	0.336
Age	1.06	1.01, 1.11	*0.023
Sex	1.49	0.46, 4.78	0.498

* p < 0.05 ; Cox proportional hazards regression

P2X7R gene expression data from high throughput qPCR and the corresponding overall survival was obtained from 29 patients with high-grade glioma. Overall survival was measured from the date of tumour resection to the date of death or last follow-up. Patient age and sex were also taken into account

### P2X7R is upregulated in the glioma microenvironment and its expression correlated with the expression of VEGFB, IL-4, MMP-9, PCNA and IL-8

P2X7R expression was subsequently correlated with the expression of the various downstream mediators as well as tumour stemness markers present in the glioma microenvironment. A pairwise Pearson correlation analyses was used to compare the gene expression of P2X7R with each of the previously measured mediators/markers: tumour and glioma stemness markers – GFAP, IDH1/2, NANOG, SOX2, SOX2-OT and CD133; tumour-promoting mediators – IL-1β, IL-4, IL-6, IL-8, IL-10, IL-13, TGF-β, TNF, VEGFA/B, EGFR, MMP-9, HIF-1α, NF-κB and PCNA; tumour-suppressing mediators—IFN-α, IFN-β, IFN-γ, IL-2 and IL-12A/B; and immune cell markers—CD68, CD45, ARG1, CD14, S100A8/9, HLADR, FOXP3 and NLRP3 (Table 3).

**Fig. 5:**
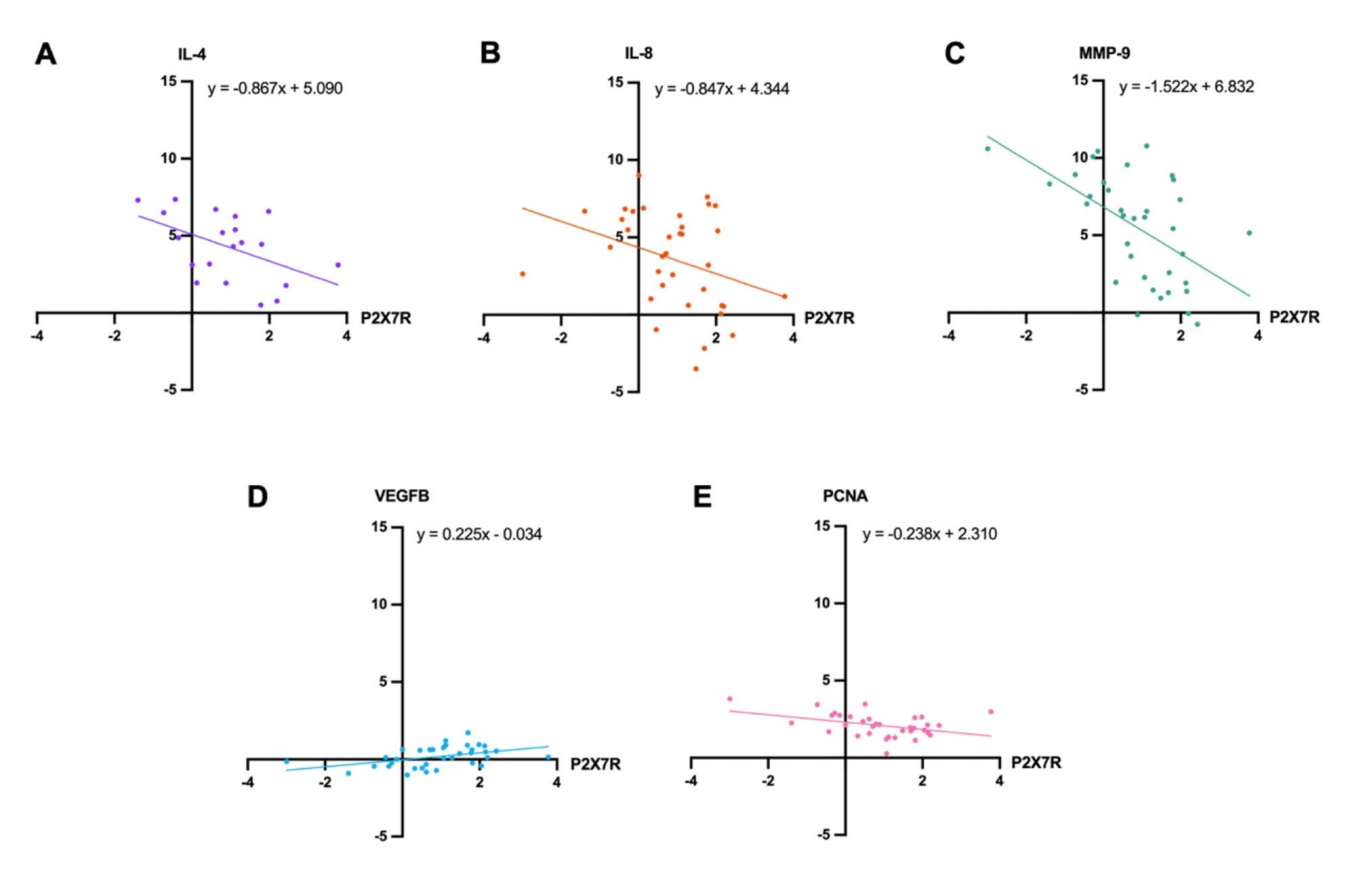
Simple linear regressions assessing the relationship between P2X7 receptor (P2X7R) and interleukin (IL)-4, IL-8, matrix metalloproteinase-9 (MMP-9), vascular endothelial growth factor B (VEGFB) and proliferative cell nuclear antigen (PCNA). Gene expression data was obtained from high throughput qPCR analyses using the Biomark 48.48 Dynamic Array Integrated Fluidic Circuits (IFC) system from Fluidigm. The fold change (FC) in gene expression relative to control was calculated using the 2^− ΔΔCt^ method for qPCR analysis. Data were transformed into log2 FC (LFC) prior to undergoing simple linear regression. P2X7R expression (LFC) served as the independent variable for all regressions. Regressions were performed based on significant correlations from a Pearson’s correlation analysis. **A** Simple linear regression between P2X7R and IL-4. R^2^ = 0.24, *p* = 0.029; b1=-0.867 ± 0.365, 95% CI=-1.634 to -0.099; b0 = 5.09 ± 0.551, 95% CI = 3.933 to 6.247. **B** Simple linear regression between P2X7R and IL-8. R^2^ = 0.11, *p* = 0.047; b1 = 0.847 ± 0.41, 95% CI=-1.681 to -0.012; b0 = 4.344 ± 0.626, 95% CI = 3.071 to 5.617. **C** Simple linear regression between P2X7R and MMP-9. R^2^ = 0.30, *p* = 0.0006; b1=-1.522 ± 0.403, 95% CI=-2.342 to -0.702; b0 = 6.832 ± 0.615, 95% CI = 5.581 to 8.083. **D** Simple linear regression between P2X7R and VEGFB. R^2^ = 0.19, *p* = 0.01; b1 = 0.225 ± 0.082, 95% CI = 0.058 to 0.393; b0=-0.034 ± 0.126, 95% CI=-0.29 to 0.221. **E** Simple linear regression between P2X7R and PCNA. R^2^ = 0.16, *p* = 0.017; b1=-0.238 ± 0.095, 95% CI=-0.431 to -0.045; b0 = 2.31 ± 0.145, 95% CI = 2.016 to 2.605

Increased P2X7R expression was found to significantly correlate with the expression levels of 5 ‘tumour-promoting’ mediators – VEGFB, IL-4, MMP-9, PCNA and IL-8. Specifically, P2X7R was moderately positively correlated with increased VEGFB expression (*r* = 0.43, *p* = 0.01, 95% CI = 0.11 to 0.67). P2X7R expression was also significantly moderately negatively correlated with IL-4 (*r*=-0.49, *p* = 0.03, 95% CI=-0.77 to -0.06), MMP-9 (*r*=-0.55, *p* = 0.0006, 95% CI=-0.75 to -0.27), and PCNA (*r*=-0.4, *p* = 0.02, 95% CI=-0.65 to -0.08). There was also a significant weak negative correlation between P2X7R expression and IL-8 (*r*=-0.34, *p* = 0.047, 95% CI=-0.60 to -0.01). Univariate linear regressions were subsequently conducted to further delineate the relationship between P2X7R (independent variable) and VEGFB, IL-4, MMP-9, PCNA and IL-8 (Fig. 5). The overall regressions for all outcome genes were statistically significant, confirming that P2X7R expression had an effect on VEGFB (*p* = 0.01, 95% CI = 0.06 to 0.39); IL-4 (*p* = 0.03, 95% CI 1.63 to 0.10), MMP-9 (*p* = 0.0006, 95% CI=-2.34 to -0.70), PCNA (*p* = 0.02, 95% CI=-0.43 to -0.05) and IL-8 (*p* = 0.047, 95% CI=-1.689 to -0.01). Taken together, these data demonstrate that significant P2X7R expression in high-grade glioma samples was positively associated with VEGFB expression, and inversely associated with IL-4, IL-8, MMP-9 and PCNA, demonstrating a potentially pro-tumour angiogenic capacity.

**Table 3:** Pearson’s Correlation of P2X7 receptor (P2X7R) expression and the expression of selected genes involved in tumour classification and glioma cell stemness, immunity and inflammation in human high-grade glioma samples

Gene (vs. P2X7R)	Pearson *r*				95% CI	*p*-value
*Glioma cell stemness markers*						
GFAP	0.19				-0.15, 0.49	0.270
IDH1	-0.24				-0.53, 0.10	0.159
IDH2	0.22				-0.13, 0.51	0.211
NANOG	-0.20				-0.50, 0.14	0.241
SOX2	-0.03				-0.36, 0.31	0.879
SOX2-OT	-0.25				-0.54, 0.09	0.145
CD133	-0.09				-0.41, 0.25	0.604
*‘Tumour-suppressing’ mediators*						
IFN-α	-0.23				-0.52, 0.11	0.183
IFN-β	-0.25				-0.54, 0.09	0.152
IFN-γ	0.01				-0.36, 0.39	0.944
IL-2	-0.36				-0.70, 0.11	0.129
IL-12A	0.16				-0.18, 0.47	0.360
IL-12B	-0.07				-0.48, 0.38	0.774
*‘Tumour-promoting’ mediators*						
IL-1β	-0.01				-0.34, 0.33	0.961
IL-4	**-0.49**				**-0.77, -0.06**	*0.029
IL-6	-0.04				-0.37, 0.29	0.803
IL-8	**-0.34**				**-0.60, -0.01**	*0.047
IL-10	-0.04				-0.37, 0.30	0.823
IL-13	-0.18				-0.57, 0.28	0.444
TGF-β	-0.26				-0.54, 0.08	0.134
TNF	0.12				-0.23, 0.44	0.493
VEGFA	-0.26				-0.55, 0.08	0.125
VEGFB	**0.43**				**0.11, 0.67**	**0.010
EGFR	-0.28				-0.56, 0.05	0.099
MMP9	**-0.55**				**-0.75, -0.26**	***0.0006
HIF-1α	-0.09				-0.41, 0.25	0.612
NF-κB	-0.01				-0.34, 0.32	0.952
PCNA	**-0.40**				**-0.65, -0.08**	*0.017
*Immune cell markers*						
CD68	-0.03				-0.36, 0.31	0.868
CD45	-0.08				-0.40, 0.26	0.663
ARG1	-0.26				-0.55, 0.08	0.136
CD14	-0.19				-0.49, 0.15	0.269
S100A8	-0.25				-0.54, 0.09	0.140
S100A9	-0.22				-0.51, 0.13	0.215
HLADR	-0.13				-0.44, 0.22	0.466
FOXP3	0.14				-0.20, 0.45	0.424
NLRP3	0.12				-0.22, 0.44	0.492

Significantly correlated genes are highlighted in bold

* p < 0.05; ** p < 0.001; *** p<0.0001; Pearson correlation

## Discussion

The median survival for patients diagnosed with glioblastoma remains under 2 years in contrast to the improvements in therapies and survival for many other cancer types [14]. While much of the complex glioma microenvironment has been characterised, studies expounding the associations between P2X7R expression and various cytokines/chemokines, immune cell markers, and glioma stemness are lacking. Hence, this study characterises the expression of P2X7R and correlates this with changes in various glioma cell stemness markers, tumour and immune cell mediators in human high-grade gliomas relative to non-tumour post-mortem brain samples. The results demonstrated the array of complex changes in these mediators in glioblastoma tissue relative to healthy controls. P2X7R expression was significantly increased in glioblastoma samples compared to postmortem healthy control brain tissue.

### Glioma classification and glioma cell stemness markers

Glioma classification and glioma cell stemness markers were universally upregulated in glioma patients compared to the controls. Significant increases in gene expression of GFAP (4-fold), a type III intermediate filament in astrocytes, and IDH1 (5-fold) were observed in tumour samples, with no observed changes in IDH2 expression (Fig. 2A). Our data are in line with previous research demonstrating overexpression of wild-type IDH1, but not IDH2, in glioblastoma [15]. IDH mutations are commonly associated with improved overall survival and progression-free survival in glioblastomas [16] whilst secondary glioblastomas without mutations in IDH1 commonly had mutations in IDH2 [17]. Considering glioblastoma is specifically defined as having wild-type IDH1, the upregulation of IDH1 but not IDH2 in this study is likely due to increased mutant IDH2 expression over wild-type IDH2 normally seen in the presence of wild-type IDH1. The expression of IDH-mutant alleles or whether patients were diagnosed with primary or secondary glioblastoma was not specifically assessed and this should be considered in subsequent studies.

The expression of various glioma stemness markers – NANOG (4-fold increase), SOX2 (3-fold increase), SOX2-OT (15-fold increase) and CD133 (3-fold increase) – were also significantly increased in high-grade glioma samples compared to the controls (Fig. 2A). The results suggest the presence of glioblastoma stem cells (GSCs) within the glioma microenvironment [18]. GSCs are a subpopulation of undifferentiated, multipotent glioma cells that asymmetrically differentiate into the heterogeneous tumour cell population and further resident GSCs [18]. These cells crucially promote glioma malignancy by fuelling resistance to treatment and tumour recurrence [18]. Hence, it was foreseeable that GSC markers were upregulated in high-grade gliomas.

### Tumour-promoting mediators and immune cells

Mediators that have previously been reported to be ‘tumour-promoting’ in a variety of settings were significantly upregulated within the glioma microenvironment of the patient cohort, compared to non-tumour brain specimens (Fig. 2C). Increased gene expressions of inflammatory mediators (MMP-9, HIF-1α and NF-κB) and growth factors/receptors (VEGFA and EGFR) were also observed, which are consistent with the current literature (Fig. 2C). The necrotic glioma microenvironment which is exacerbated by neuroinflammation, generates a hypoxic environment where the ability of glioma cells to adapt and thrive is mediated by HIF-1α, which is upregulated in glioblastoma [4, 19]. HIF-1α overexpression in gliomas is also driven by the activation of EGFR via the PI3K pathway, with EGFR gene amplification being a demonstrated hallmark of glioblastoma that contributes to gliomagenesis [19, 20]. Notably, HIF-1α is also a potent activator of VEGF, which is also overexpressed in glioblastoma and serves as the predominant inducer of angiogenesis in gliomas [19]. VEGF promotes the formation of new abnormal vasculature essential for exponential tumour growth [21]. NF-κB was also upregulated in patient glioma samples compared to controls. Upregulation and constitutive activation of NF-κB in glioblastoma has been well demonstrated, with its effects mediating the activation of genes associated with cell cycle regulation, inhibition of apoptosis, inflammation and cell adhesion [22]. In addition, there was an aberrant 44-fold increase of MMP-9 in the glioma microenvironment when compared to healthy controls. MMP-9 is a proteolytic enzyme involved in extracellular matrix remodelling and degradation. It is upregulated in gliomas, with its expression being positively correlated with poor prognosis [7].

Notably, there was overexpression of various cytokines, including IL-1β, IL-4, IL-6, IL-8, IL-10, TGF-β and TNF in the patient cohort (Fig. 2C), which was unsurprising given that these cytokines serve primarily immunosuppressive roles in the glioma microenvironment [4]. The most amplified cytokine was TNF, which was observed to be increased by almost 50-fold compared to control samples. In gliomas, TNF release induces neovascularisation via VEGF and T cell depletion via the activation of immunosuppressive macrophages [4]. TNF activation also induces the transcription of IL-1, IL-6 and IL-8 [4]. IL-1, including IL-1β, potently induces tumour angiogenesis and invasion by promoting the release of VEGF and MMPs by glioma cells as well as the polarisation and recruitment of immunosuppressive TAMs [23, 24]. Both IL-6 and IL-8 also contribute to glioma growth by promoting angiogenesis, tumour proliferation and resistance to apoptosis [4].

Interestingly, IL-13 expression was significantly downregulated in patient glioma samples compared to the controls (Fig. 2C). Few studies have characterised IL-13 expression in gliomas with the majority of research focused on the IL-13 receptor subunit alpha-2 (IL-13Rα2), which was shown to be aberrantly expressed in almost 80% of glioblastoma samples [25]. IL-13 itself has been reported to inhibit the anti-tumour response by downregulating CD8+ T cell immunosurveillance [26], although this study was not specific to gliomas. Hence further characterisation of the role and expression of IL-13 in gliomas is much needed.

### Tumour-suppressing mediators

The expression of various previously described ‘tumour-suppressing’ mediators were also quantified in human glioma samples compared to non-tumour brain specimens. Although various tumour-suppressing mediators were upregulated relative to the control, their level of expression (FC) were to a lesser extent than ‘tumour-promoting’ mediators (Fig. 2B). Notably, IFN-α and IFN-β expression were increased by approximately 3-fold in human glioma samples, versus healthy controls. IFN-α induces both cell cycle arrest and apoptosis, while IFN-β promotes CD8+ T cell-mediated glioma cytotoxicity [4]. Amplified IL-12A and IL-12B gene expression profiles were also observed in human high-grade glioma samples compared to the controls. IL-12 retains its anti-tumour function in the glioma microenvironment by enhancing CD8+ T cell cytotoxicity [27]. Given the inflammatory nature of the tumour microenvironment, increases in both tumour-promoting and tumour-suppressing mediators are expected; however, the imbalance in favour of immunosuppression overrides anti-tumour activity.

### P2X7R expression and its link to glioma classification and GSC markers, inflammatory mediators and immune cells, and patient survival

P2X7R stimulation has been extensively linked to neuroinflammation [28, 29], although its roles in glioma-associated inflammation, immune cells and glioma cells require further clarification. Indeed, P2X7R expression was significantly increased in the patient cohort (Fig. 4A), which is consistent with previous reports of P2X7R upregulation in gliomas compared to expression in normally healthy brain tissue [10, 30]. Furthermore, increased P2X7R expression has also been specifically associated with increasing glioma grades (I, II, III, & IV), suggesting P2X7R is potentially associated with tumour progression [31], and that its expression levels could be used as a marker of disease severity. Whether the level of the receptor being higher in higher grade gliomas is a casual association or is secondary to progression is unclear. Elevated HIF-1α levels in the glioma microenvironment are known to contribute to increased P2X7R expression, since hypoxic conditions increase extracellular ATP levels, and P2X7R is activated exclusively in high-ATP environments [32, 33]. Receptor activation further initiates the production of various inflammatory mediators. Limited studies have assessed the relationship between P2X7R and the markers of inflammation (cytokines/chemokines, immune cell markers) in patient-derived high-grade glioma samples, compared to healthy brain. Of the 38 genes assessed, increased P2X7R expression was found to be significantly associated with increased VEGFB expression, but decreased IL-4, IL-8, MMP-9 and PCNA expression (Table 3; Fig. 5). This data aligns with the receptor’s current associations with VEGF. For instance, P2X7R blockade diminished VEGF levels in a HIF-1α-dependent manner, which hindered neuroblastoma progression [13]. P2X7R-mediated VEGFB secretion might also contribute to glioma angiogenesis by boosting VEGFA activity [34]. VEGFB is also known to directly promote cell survival by preventing activation of apoptosis-related genes [34]. Hence, one of the mechanisms of P2X7R-mediated glioma progression is likely via the upregulation of VEGF. On the other hand, the inverse associations between P2X7R expression and IL-4, IL-8, MMP-9 and PCNA were not in line with previous speculations of P2X7R and glioma promotion [35–37]. IL-4, IL-8, MMP-9 and PCNA promote glioma survival by inducing TAM immunosuppression, angiogenesis, glioma cell proliferation and clonogenic potential, and DNA replication and repair, respectively [4, 7, 38, 39]. P2X7R inhibition has also been reported to downregulate MMP-9 expression in macrophages, whereas P2X7R stimulation enhanced PCNA expression in gliomas [36]. P2X7R activation also facilitates IL-8 release from eosinophils during inflammation [11]. Studies on eosinophil involvement in the glioma microenvironment are scarce, but there is some evidence of eosinophil recruitment to necrotic tissue in a murine model of glioblastoma [40]. Collectively, few links have been drawn between P2X7R and IL-4, IL-8, MMP-9 and PCNA. Given the tumour-trophic associations between these mediators and P2X7R in glioma, decreased expression of IL-4, IL-8, MMP-9 and PCNA maybe compensatory in nature. Alternatively, considering the complex and heterogeneous nature of the glioblastoma microenvironment, the association demonstrated of course does not indicate causation. Future studies should utilise assays to investigate any possible direct relationship between P2X7R and these mediators.

Finally, since there was significant P2X7R overexpression in the patient cohort, a link between P2X7R expression and overall glioma survival was investigated. Both the Kaplan-Meier analysis and the Cox proportional hazards regression yielded no significant association between P2X7R expression and overall survival (Fig. 4B; Table 2). Studies on P2X7R expression and glioma survival are limited and present conflicting results. However, data from the survival analyses are in line with a recent study conducted by Matyśniak et al. [41]. In the study, they observed significant downregulation of negative prognostic cancer markers following P2X7R antagonism [41]. The authors subsequently conducted survival analyses assessing the link between P2X7R expression and overall survival from a cohort of 131 glioblastoma patients from the TCGA database. No significant association between P2X7R expression and glioblastoma survival was observed [41]. To note, only patients whose survival time was greater than 100 and less than 1000 days were selected. Imposing restrictions on survival time introduces a significant level of bias to the analyses, reducing the overall validity of their data. On the contrary, an earlier investigation headed by Gehring and colleagues demonstrated a significant survival advantage in glioma patients with high P2X7R expression [42]. Results were consistent across Kaplan-Meier analyses conducted on 18 human glioma samples and on data obtained from 343 glioma patients in the REMBRANDT public database. In the same study, P2X7R survival curves of 47 patients with gliomas in the database revealed improved response to therapy in patients with high P2X7R expression, quantified by increased median survival times. However, this study was not restricted to high-grade gliomas [42], unlike our current study. The relationship between P2X7R expression and overall survival in high-grade gliomas remains inconclusive and should be investigated with a larger patient cohort in the near future.

## Conclusion

Collectively, data from this investigation provides detailed characterisation of the expression (FC) of P2X7R along with various glioma cell stemness markers, tumour and immune cell mediators in high-grade gliomas compared to non-tumour post-mortem brain controls. The overexpression of various genes related to both tumour-promotion and tumour-suppression is consistent with the complex multidirectional neuroinflammatory milieu exhibited in glioblastoma. However, there was more extensive amplification of pro-tumourigenic genes, in favour of a predominantly tumour-proliferative and immunosuppressive environment. Importantly, this study confirmed significant elevation of P2X7R in human high-grade gliomas, compared to controls. There was also an over-abundance of innate immune cells in the glioma microenvironment, compared to non-tumour samples, highlighting potential crucial roles for monocytes, microglia, and macrophages in glioma pathogenesis. However, no links between P2X7R expression, the inflammatory molecular phenotype and overall survival were established, prompting the need for further investigation into their pathophysiological and clinical significance for patients with glioblastomas.

## Data Availability

The raw data supporting the conclusions of this article will be made available by the authors, without undue reservation.

## Abbreviations

BBB: Blood–brain barrier
CD: Cluster of differentiation
CI: Confidence Interval
CNS: Central nervous system
Ct: Cycle threshold
EGFR: Epidermal growth factor receptor
FC: Fold change
GFAP: glial fibrillary acidic protein
GSC: Glioma stem cell
HIF: 1–Hypoxia–inducible factor–1
HR: Hazard ratio
HREC: Human Research Ethics Committee
IDH: Isocitrate dehydrogenase
IFC: Integrated fluidic circuits
IL: Interleukin
IL-1β: Interleukin–1–beta
INF: Interferon
LFC: Log2 fold change
MHTP: Monash Health Translation Precinct
MMP: Matrix metalloproteinase
NF-κB: Nuclear factor–kappa B
NLRP3: NLR family pyrin domain containing 3
P2X7R: Purinergic P2X receptor 7
PI3K: Phosphoinositide 3–kinase
RMH: Royal Melbourne Hospital
SOX2: SRY–box 2
SOX2: OT–SOX2 overlapping transcript
TAM: tumour–associated microglia/macrophages
TGF: β–Transforming growth factor–beta
VEGF: Vascular endothelial growth factor
